# Molecular Mechanisms Underlying the Establishment and Maintenance of Vascular Stem Cells in *Arabidopsis thaliana*

**DOI:** 10.1093/pcp/pcac161

**Published:** 2022-11-18

**Authors:** Shunji Shimadzu, Tomoyuki Furuya, Yuki Kondo

**Affiliations:** Department of Biology, Graduate School of Science, Kobe University, 1-1 Rokkodai, Kobe, 657-8501 Japan; Department of Biological Sciences, Graduate School of Science, The University of Tokyo, 7-3-1 Hongo, Bunkyo-Ku, Tokyo, 113-0033 Japan; Department of Biology, Graduate School of Science, Kobe University, 1-1 Rokkodai, Kobe, 657-8501 Japan; College of Life Sciences, Ritsumeikan University, 1-1-1 Noji-higashi, Kusatsu, 525-8577 Japan; Department of Biology, Graduate School of Science, Kobe University, 1-1 Rokkodai, Kobe, 657-8501 Japan

**Keywords:** Regulatory network, Stem cell, Vasculature, VISUAL

## Abstract

The vascular system plays pivotal roles in transporting water and nutrients throughout the plant body. Primary vasculature is established as a continuous strand, which subsequently initiates secondary growth through cell division. Key factors regulating primary and secondary vascular developments have been identified in numerous studies, and the regulatory networks including these factors have been elucidated through omics-based approaches. However, the vascular system is composed of a variety of cells such as xylem and phloem cells, which are commonly generated from vascular stem cells. In addition, the vasculature is located deep inside the plant body, which makes it difficult to investigate the vascular development while distinguishing between vascular stem cells and developing xylem and phloem cells. Recent technical advances in the tissue-clearing method, RNA-seq analysis and tissue culture system overcome these problems by enabling the cell-type-specific analysis during vascular development, especially with a special focus on stem cells. In this review, we summarize the recent findings on the establishment and maintenance of vascular stem cells.

## Introduction

During tissue and organ development in plants, cell division and cell fate specification occur in the meristems. The division of cells in shoot and root apical meristems contributes to increasing the height and branch number of the plant body, while secondary radial growth contributes to increasing the plant thickness. Secondary growth mainly results from permanent rounds of cell division in the secondary meristem, cambium, which is located within the vascular tissues. Plant meristems contain stem cells, which are defined as cells that have the capacity to self-renew and the potential to differentiate into several specialized cell types. Although the regulatory mechanisms of meristems have been well studied, studies on plant stem cells are limited.

Vasculature is the main transport system of plants and is composed of the xylem, which is responsible for the transport of water and nutrients; phloem, which is responsible for the transport of metabolites; and cambium. The cambium consists of multiple cell layers, one of which has recently been identified as the layer of vascular stem cells (Shi et al. 2019, Smetana et al. 2019). Vascular stem cells are initially developed from primary meristems and subsequently activate their division capability, which triggers the onset of secondary growth. During secondary growth, stem cells give rise to xylem progenitor cells and phloem progenitor cells while maintaining themselves by proliferation. To ensure continuous radial growth, the maintenance of vascular stem cells should be strictly regulated. Recent genetic analyses of the model plant *Arabidopsis thaliana* led to the identification of the key regulators of the cambium (reviewed in Hoang et al. 2020, Haas et al. 2022). Omics approaches with a cell-type-specific resolution are gradually unveiling the nature of vascular stem cells. In this review, we summarize the regulatory mechanisms underlying the establishment, maintenance and differentiation of vascular stem cells, with a special focus on phytohormones, cell–cell interactions and gene regulatory networks.

## Development of Vascular Cells in Primary Growth

**Fig. 1:**
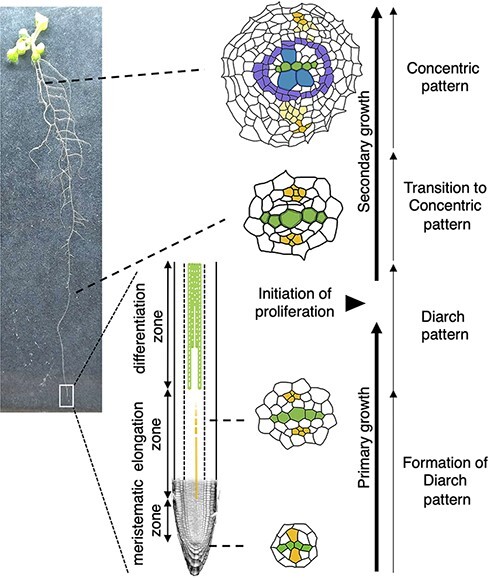
Transition from primary vascular development to secondary development. Schematic illustrations of the cross-sections of vasculature from the root tip to the hypocotyl. Green, yellow, blue and purple colors indicate the primary xylem, primary phloem, secondary xylem and cambium, respectively.

**Fig. 2:**
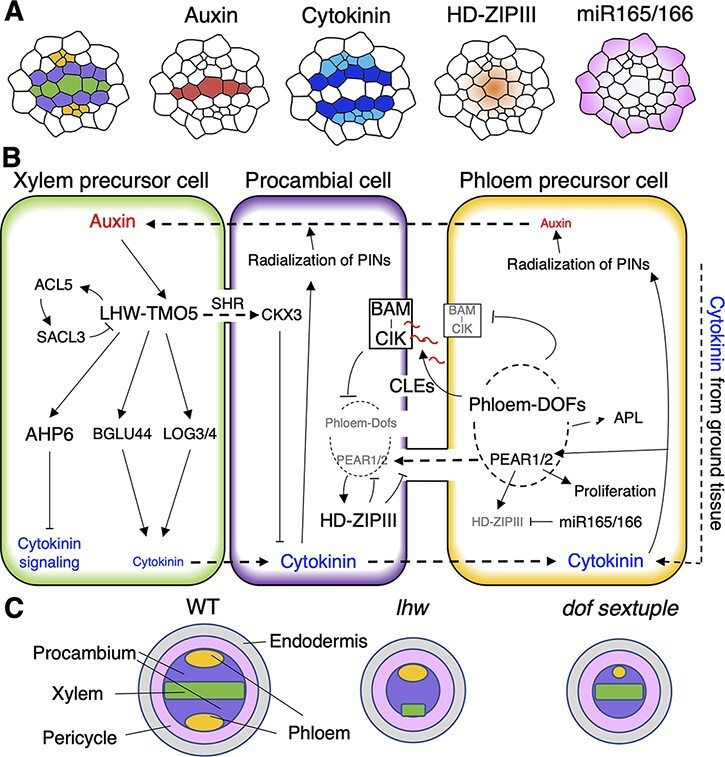
Molecular mechanisms regulating primary vascular patterning. (A) Schematic showing the hormone response or gene expression patterns in the primary vasculature. (B) Schematic of the interaction networks involving key regulatory factors in the xylem precursor cell, procambial cell and phloem precursor cell. (C) Schematic showing the phenotype of mutants defective in primary growth (Wildtype (WT), *lhw* and *dof sextuple*). Green, yellow, purple, pink and gray colors indicate the primary xylem, primary phloem, procambium, pericycle and endodermis, respectively.

In Arabidopsis roots, vascular cells originate from vascular initial cells located immediately above the quiescent center (**Fig. 1**). Vascular initial cells give rise to vascular precursor cells and push them out toward the basal part of roots. These vascular precursor cells undergo further anticlinal–horizontal divisions in the root meristem: anticlinal–horizontal divisions to generate vascular cells along the longitudinal plane and periclinal divisions to increase the cell number along the cross-sectional plane (**Fig. 1**). During this developmental process, a xylem axis and two phloem poles are properly established in the vasculature. The xylem vessel cells are arranged in a central row, and two phloem poles are formed in a symmetrical position across the xylem axis. Procambial cells are positioned between the xylem and phloem cells (**Fig. 1**). Numerous previous studies have revealed that mutually inhibitory regulation between two phytohormones, auxin and cytokinin, plays an important role in such diarch pattern formation. A high auxin response domain is formed along the central xylem axis, whereas a high cytokinin response domain is formed in the surrounding procambial cell region (Bishopp et al. 2011a) (**Fig. 2**). Cytokinin synthesized in shoots travels to the root tip via the phloem to induce the expression of genes encoding PIN-FORMED (PIN) auxin efflux carriers. Cytokinin also promotes the lateral polar localization of PIN, thereby enabling the accumulation of auxin in the xylem axis (Mähönen et al. 2000, Bishopp et al. 2011a, 2011b). In addition, mobile transcription factors encoded by *AT-HOOK MOTIF NUCLEAR LOCALIZED PROTEIN 3* (*AHL3*) and *AHL4* genes, which are upregulated in response to cytokinin in the procambium, move intercellularly to further maintain the boundary between the two hormonal domains (Zhou et al. 2013). On the other hand, in the xylem axis, the expression of basic helix-loop-helix (bHLH) genes, *TARGET OF MONOPTEROS 5* (*TMO5*) and its homolog *TMO5-LIKE1* (*T5L1*), is induced by auxin. TMO5 and T5L1 form a heterodimeric complex with LONESOME HIGHWAY (LHW), an atypical bHLH transcription factor, to promote periclinal cell divisions in the root vasculature. Indeed, the loss-of-function mutation of *LHW* decreases the number of vascular cells and disrupts the diarch patterning (**Fig. 2**) (De Rybel et al. 2014, Ohashi-Ito et al. 2014). The LHW–TMO5 complex upregulates the expression of cytokinin biosynthesis genes, *LONELY GUY 3* (*LOG3*) and *LOG4*, in xylem precursor cells (**Fig. 2**). The synthesized cytokinin promotes the division in procambial cells in a non-cell-autonomous manner partly through one of the DNA-BINDING WITH ONE FINGER (DOF) transcription factors, Dof2.1 (Smet et al. 2019). In addition, the LHW–TMO5 complex also induces the transcription of *ARABIDOPSIS HISTIDINE PHOSPHOTRANSFER PROTEIN 6* (*AHP6*), a negative regulator of cytokinin signaling (Mähönen et al. 2006), to prevent the elevation of cytokinin signaling in xylem precursor cells. Furthermore, single-cell RNA-sequencing (RNA-seq) analysis of root tips revealed new downstream components of the LHW–TMO5 complex, namely B-S GLUCOSIDASE 44 (BGLU44) and CYTOKININ OXIDASE/DEHYDROGENASE 3 (CKX3). *BGLU44* is expressed in xylem precursor cells and is involved in cytokinin biosynthesis, like *LOG4*, whereas *CKX3* is expressed in the adjacent procambial cells and functions to degrade cytokinin. *CKX3* upregulation requires the translation and intercellular movement of SHORT ROOT proteins. Therefore, cytokinin synthesis by BGLU44 and subsequent cytokinin degradation by CKX3 occur downstream of the LHW–TMO5 complex with a time lag, enabling the spatiotemporal control of cytokinin signaling in the vasculature (**Fig. 2**) (Yang et al. 2021). In addition, the LHW–TMO5 complex induces the expression of the thermospermine synthase gene, *ACAULIS 5* (*ACL5*), thereby activating the translation of the bHLH transcription factor SUPPRESSOR OF ACAULIS5 LIKE 3 (SACL3). SACL3 is able to heterodimerize with LHW and its homologs as well as with TMO5, thus competitively inhibiting the formation of the LHW–TMO5 complex. In other words, the LHW–TMO5 complex forms a negative feedback circuit through ACL5 and SACL3 for the robust control of cell division in the procambium (**Fig. 2**) (Katayama et al. 2015, Vera-Sirera et al. 2015).

In protophloem sieve elements (PSEs), six members of the DOF transcription factor family have been recently reported to act downstream of cytokinin to regulate periclinal cell divisions (Miyashima et al. 2019). Among these DOF transcription factors, PHLOEM EARLY DOF 1 (PEAR1; Dof2.4) and PEAR2 (Dof5.1) translocate from PSEs to neighboring cells through plasmodesmata to promote cell division in and around PSEs. Mobile PEAR1 and PEAR2 induce the expression of Arabidopsis *CLASS III HOMEODOMAIN-LEUCINE ZIPPER* (*HD-ZIP III*) transcription genes including *HOMEOBOX 8*, *PHABULOSA*, *PHAVOLUTA*, *REVOLUTA* and *CORONA*. However, these HD-ZIP III transcription factors inhibit cell division by suppressing the *PEAR* gene expression and restricting the PEAR protein movement, thus constituting a negative feedback loop. On the other hand, HD-ZIP IIIs are negatively affected by the out–in gradient of miR165/166 in the stele (Carlsbecker et al. 2010), resulting in a robust boundary between dividing and nondividing cells in the vasculature (**Fig. 2**) (Miyashima et al. 2019). Besides, DOF transcription factors contribute to the positive regulation of PSE differentiation in a cell-autonomous manner (Roszak et al. 2021, Qian et al. 2022). DOF transcription factors activate their gene expression via a positive feedback loop, eventually inducing the expression of the master regulator of phloem differentiation, *ALTERED PHLOEM DEVELOPMENT* (*APL*) (Bonke et al. 2003, Roszak et al. 2021, Qian et al. 2022). DOFs also induce genes encoding secretory peptides such as CLAVATA3/EMBRYO SURROUNDING REGION–related 25 (CLE25), CLE26 and CLE45, which inhibit PSE formation (Depuydt et al. 2013, Rodriguez-Villalon et al. 2014, Hu, C. et al., 2022). CLE peptides suppress the *DOF* gene expression to prevent excess sieve element (SE) differentiation (**Fig. 2**) (Qian et al. 2022). Simultaneously, the secreted CLE peptides inhibit PSE differentiation in more premature cells in the same cell file as autocrine signals (Depuydt et al. 2013, Rodriguez-Villalon et al. 2014). Consistent with these results, the DOF sextuple mutant has reduced cell number and defects in phloem formation (**Fig. 2**). Taken together, the establishment of cellular patterning during primary vascular development is properly controlled through complex intercellular communication with phytohormones, secreted peptides and mobile proteins.

## Establishment of the Cambium for Secondary Growth

After completing primary vascular patterning, procambial cells and vasculature-surrounding pericycle cells undergo periclinal divisions. This event marks the start of secondary growth, which occurs in the upper part of the roots. Procambial and cambial cells also give rise to secondary xylem cells and secondary phloem cells toward the inner and outer sides, respectively. Cambium is a ring-shaped meristematic tissue formed between the xylem and phloem tissues. Recent clonal analysis with the Cre-*LoxP* recombination system revealed that only procambial cells and pericycle cells adjacent to the primary xylem cells contribute to the formation of the vascular cambium (Smetana et al. 2019). In this process, cytokinin functions to trigger the division of procambial cells. The *atipt1;3;5;7*, cytokinin biosynthesis mutant exhibits no secondary growth, which is rescued by the application of cytokinin (Matsumoto-Kitano et al. 2008). In response to cytokinin, *LOB DOMAIN-CONTAINING PROTEIN 3* (*LBD3*) and *LBD4* are rapidly induced, which are required for the activation of secondary growth. Subsequently, expression levels of *LBD1* and *LBD11* are upregulated with a delay and then are kept during secondary growth. These LBD transcription factors are considered to repress cytokinin signaling, thus forming a negative feedback loop for controlling secondary growth (Ye et al. 2021).

**Fig. 3:**
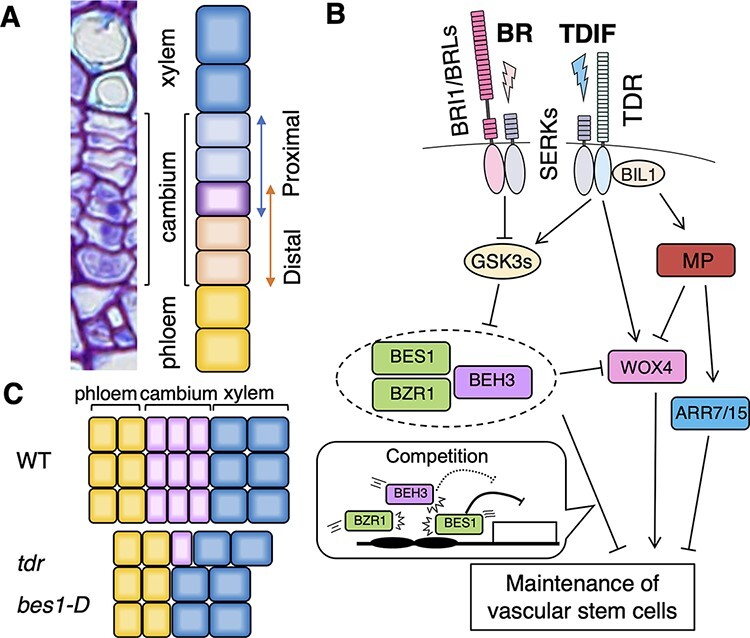
Pathways regulating vascular stem cell maintenance. (A) A picture and schematic illustration of vascular tissues: xylem, proximal cambium (xylem side), distal cambium (phloem side) and phloem. Purple color indicates a vascular stem cell. (B) Schematic of signaling pathways regulating the maintenance of vascular stem cells. (C) Schematic showing the phenotype of mutants defective in secondary growth (WT, *tdr* and *bes1-D*).

In the vasculature, the cambium consists of multiple cell layers. Sanio postulated the existence of vascular stem cells in the cambium in 1873, based on histological analyses (Sanio 1873). According to this theory, vascular stem cells are arranged as a single-cell file in the cambium layers and alternately produce xylem and phloem progenitors. Difficulties in live imaging, because of the deep location of the vascular tissue, kept this theory a mystery for a long time; however, recent clonal analyses support the existence of bifacial vascular stem cells in the cambium (**Fig. 3**) (Bossinger and Spokevicius 2018, Shi et al. 2019, Smetana et al. 2019). Smetana et al. (2019) showed that xylem precursor cells act as organizers and convert the adjacent cells into vascular stem cells. In Arabidopsis roots, a local maximum of auxin signaling is observed on the xylem side (proximal) of the vascular cambium, which promotes the expression of *HD-ZIP III* genes through MONOPTEROS (MP)/AUXIN RESPONSE FACTOR 5 (ARF5) and other ARF transcription factors. *HD-ZIP III* genes promote xylem identity in the xylem precursor and confer stem cell identity to the adjacent cells in a non-cell-autonomous manner to induce cell division. Once the organizer cell differentiates into a xylem vessel, one of the stem cell’s daughter cells on the xylem side becomes a new organizer, while the other daughter cell is maintained as a vascular stem cell (Smetana et al. 2019). This model accounts for the continuous cycle of stem cell division and commitment but does not fully explain what initiates the establishment of stem cell identity, because primary xylem cells undergo programmed cell death at a much earlier developmental stage than the initiation of secondary growth. Further studies connecting the role of cytokinin and auxin are needed to understand vascular stem cell establishment.

## Maintenance of Vascular Stem Cells during Secondary Growth

During secondary growth, vascular stem cells continuously give rise to xylem and phloem cells while maintaining themselves by self-renewal. Therefore, vascular stem cells must be maintained permanently. Tracheary element differentiation inhibitory factor (TDIF), a member of the CLE peptide hormone family, is known as one of the major regulators of vascular stem cell maintenance. The 12-amino-acid TDIF peptide was originally isolated from the culture medium of a tracheary element differentiation induction system with *Zinnia elegans* as a TDIF (Ito et al. 2006). In Arabidopsis, TDIF is secreted from phloem cells and is received by the receptor protein TDIF RECEPTOR (TDR)/PHLOEM INTERCALATED WITH XYLEM (PXY) in the cambium (Fisher and Turner 2007, Hirakawa et al. 2008, Morita et al. 2016). Mutants defective in TDR often lack a cambium layer between the xylem and phloem tissues, indicating that TDIF–TDR signaling plays an essential role in the maintenance of vascular stem cells (**Fig. 3**) (Hirakawa et al. 2010). TDR localizes to the plasma membrane and functions together with co-receptors BRASSINOSTEROID INSENSITIVE 1 (BRI1)-ASSOCIATED RECEPTOR KINASE 1/SOMATIC EMBRYOGENESIS RECEPTOR-LIKE KINASE 3 (BAK1/SERK3), SERK1 and SERK2 (Zhang et al. 2016a, 2016b). XYLEM DIFFERENTIATION, DISRUPTION OF VASCULAR PATTERNING (XVP)/NAC003, a NAM- / ATAF1-/CUC(NAC)-type transcription factor, is localized at the plasma membrane and interacts with the TDR–BAK1 complex to suppress TDIF signaling (Yang et al. 2020). GLYCOGEN SYNTHASE KINASE 3 PROTEINs (GSK3s), including BRASSINOSTEROID INSENSITIVE 2 (BIN2), act as the downstream components of TDIF signaling by interacting with the intracellular kinase domain of TDR. Upon the reception of TDIF, GSK3s are released from the complex with TDR and inactivate BRI1-EMS-SUPPRESSOR1 (BES1)/BRASSINAZOLE RESISTANT 1 (BZR1) family transcription factors by direct phosphorylation (Kondo et al. 2014). BES1 and its homologs promote the differentiation of vascular stem cells into xylem and phloem cells. Similar to the *tdr* mutant, a gain-of-function mutant of BES1, *bes1-d*, occasionally causes the adjacency of xylem and phloem cells (**Fig. 3**) (Kondo et al. 2014, Saito et al. 2018). Thus, the TDIF–TDR–GSK3s–BES1 signaling cascade negatively controls xylem cell differentiation. TDIF also promotes cambial cell division through another pathway, elevating the expression of *WUS-RELATED HOMOEBOX 4* (*WOX4*) and *WOX14* (Hirakawa et al. 2008, Etchells et al. 2013). Genetic analysis with mutants for GSK3s and WOX4 revealed that the TDIF–TDR–GSK3s–BES1 and TDIF–TDR–WOX4 pathways cooperatively contribute to vascular stem cell maintenance by suppressing cell differentiation and promoting cell division (**Fig. 3**) (Kondo et al. 2014). A recent study showed that BES1 directly binds to the *WOX4* promoter to repress its expression (Hu et al. 2022), suggesting the possibility that the regulation of cell differentiation and cell proliferation affects each other to ensure the maintenance of vascular stem cells.

Molecular mechanisms underlying the regulation of vascular stem cells are being investigated with a Vascular cell Induction culture System Using Arabidopsis Leaves (VISUAL). In VISUAL, Arabidopsis cotyledons are cultured in a liquid medium supplemented with auxin and cytokinin, and a chemical inhibitor of GSK3s, namely bikinin, is added to induce vascular cell differentiation ectopically. During this process, mesophyll cells in cotyledons synchronously acquire vascular stem cell–like identity and differentiate into xylem or phloem cells (Kondo et al. 2015, 2016, Saito et al. 2018). By utilizing VISUAL, we can easily evaluate the impact of plant hormones on vascular stem cell differentiation. Brassinosteroids, which regulate GSK3s and BES1 (Li and Nam 2002, Yin et al. 2002), have a promotive effect on xylem differentiation in VISUAL, which competes with the inhibitory effect of TDIF, thus balancing xylem differentiation (Kondo 2022). Moreover, the available mutants can be genetically analyzed with VISUAL. For example, VISUAL-based analysis of *bes1* loss-of-function mutants revealed the accumulation of vascular stem cells, because of the inhibition of their differentiation into xylem and phloem cells, which confirms that BES1 is required for vascular stem cell regulation (Saito et al. 2018). Arabidopsis possesses six BES/BZR homologs. BZR1, the closest and functionally redundant homolog of BES1, promotes vascular stem cell differentiation (Saito et al. 2018). However, one of the BES1/BZR1 family members, BES1/BZR1 HOMOLOG 3 (BEH3), represses vascular stem cell differentiation, in contrast to BES1 and BZR1 (Furuya et al. 2021). BEH3 exhibits a much weaker transcriptional repressor activity than other BES/BZR transcription factors, resulting in a competitive relationship among the BES/BZR family members. Interestingly, the *beh3* loss-of-function mutant exhibited a large variation in the vasculature size, which suggests that BEH3 functions to stabilize the activity of vascular stem cells by competing with other BES/BZR members.

GSK3 proteins including BIN2 are central components of the TDIF–TDR signaling pathway (Kondo et al. 2014), as described earlier. A total of 10 members of GSK3s exist in Arabidopsis. BIN2 and its closest homologs, BIN2-LIKE1 (BIL1) and BIL2, redundantly mediate brassinosteroid signaling (Yan et al. 2009; reviewed in Saidi et al. 2012). Although BIN2 and its homologs can bind to TDR, their role in TDIF signaling differs. BIN2 and BIL2 are released from TDR upon TDIF perception, resulting in the suppression of xylem cell differentiation through the inactivation of BES1. However, BIL1 does not dissociate from TDR upon TDIF perception (Kondo et al. 2014); instead, BIL1 suppresses the division of vascular stem cells by repressing cytokinin signaling through the phosphorylation of MP/ARF5 and the consequent upregulation of *ARABIDOPSIS RESPONSE REGULATOR 7* (*ARR7*) and *ARR15* (Han et al. 2018). Therefore, the robust maintenance of vascular stem cells may require the functional divergence of genes with different and sometimes opposite functions.

## Gene Regulatory Network Underlying Vascular Stem Cell Maintenance

The TDIF–TDR signaling is a major pathway regulating vascular stem cell maintenance. In fact, this pathway is reported to cross talk with various phytohormones and peptide hormones. Strigolactones, which are involved in mycorrhizal symbiosis and branching, promote the division of vascular stem cells. An F-box protein MORE AXILLARY GROWTH 2, which mediates strigolactone signaling, induces the degradation of BES1 and BZR1, thereby increasing the transcription of *WOX4* (Agusti et al. 2011, Hu et al. 2022b). It has been reported that ARF5/MP represses the transcript levels of *WOX4* via direct binding to its promoter (Brackmann et al. 2018). On the other hand, the conditional knockdown of *MP* leads to the downregulation of *WOX4* (Smetana et al. 2019). Transcription factors ETHYLENE RESPONSE FACTOR 018 (ERF018) and ERF109, which act downstream of ethylene and/or jasmonate signaling, positively regulate vascular stem cell division to compensate for the lack of the TDIF–TDR pathway (Etchells et al. 2012). Different types of CLE peptides that act in root and shoot apical meristems promote vascular cell division in a manner additive to the TDIF signaling pathway (Whitford et al. 2008). Among the other peptides, EPIDERMAL PATTERNING FACTOR-LIKE family peptides regulate vascular stem cell activity through a leucine-rich repeat (LRR)-type receptor, ERECTA (ER), located in the phloem (Uchida and Tasaka 2013). TDR/PXY and its paralogues PXY-like1 (PXL1) and PXL2 genetically interact with ER family members including ER-LIKE1 (ERL1) and ERL2 to coordinate secondary vascular development (Wang et al. 2019). Furthermore, the LRR-type receptor MORE LATERAL GROWTH1 (MOL1) negatively controls vascular stem cells by suppressing ethylene signaling independently of the TDIF–TDR pathway (Gursanscky et al. 2016), although the ligand of MOL1 remains unclear. Additionally, a recent study reported that the expression of *CLE44*, encoding TDIF, is induced in dark conditions and prevented by blue light signaling in a PHYTOCHROME INTEREACTING FACTOR (PIF)-dependent manner (Ghosh et al. 2022).

The development of omics analyses, owing to the advances in sequencing technologies in recent years, has led to a comprehensive analysis of the cambium including vascular stem cells. The TDIF–TDR signaling pathway regulates not only the maintenance of vascular stem cells but also the division plane of these cells as a positional cue (Etchells and Turner 2010). TDIF is produced in the phloem; however, when altering the gradient of TDIF through the expression of *CLE41* in the opposite side, xylem, the patterning of vascular tissue is disordered. This effect is not observed in the *lbd4* mutant, suggesting that LBD4 functions downstream of the TDIF–TDR signaling pathway to regulate vascular patterning (Etchells and Turner 2010, Smit et al. 2020). Smit et al. constructed a transcriptional regulatory network downstream of TDR by focusing on the interaction between promoter sequences and transcription factors (Smit et al. 2020). A TDR-mediated transcriptional regulatory network containing 690 transcription factor–promoter interactions was constructed using a high-throughput enhanced yeast one-hybrid assay. The network presented a WOX14–TMO6–LBD4 feedforward loop, in which WOX14 positively regulates the *LBD4* expression, either directly or through TMO6, downstream of the TDIF–TDR pathway. Zhang et al. performed fluorescence-activated cell sorting (FACS) using a fluorescent reporter line of *ARR15*, which was specifically expressed in the procambial and cambial cells of the root vasculature. The obtained transcriptome data revealed key transcription factor genes involved in secondary growth, and 13 of these genes including *WOX4, LBD4*, *KNOTTED1-LIKE HOMEOBOX GENE 1* and *PETAL LOSS* were selected to represent the core of the regulatory network, based on the vascular phenotypes of their overexpression lines (Zhang et al. 2019). This cambium transcriptional regulatory network uncovers multiple levels of hierarchical interactions that occur during vascular secondary development.

Additionally, efforts have been made to conduct comprehensive gene expression analyses of the cambium with a spatial and/or temporal resolution. Since vascular stem cells differentiate into xylem and phloem cells synchronously in VISUAL, time-course transcriptome analysis enables the dissection of the sequential cell differentiation process in a temporal manner. Previously, the microarray data of the *apl* mutants, which exhibit a defect in phloem differentiation, and the FACS data sets generated using a phloem SE marker gene, *SIEVE ELEMENT OCCLUSION RELATED 1*, were used to construct a co-expression gene network covering the early-to-late phloem SE differentiation process (Kondo et al. 2016). Furthermore, we recently integrated these data with the 6-h-interval time-course data set and transcriptome data sets of the *bes1* mutant to construct a co-expression network of the whole VISUAL differentiation process. The network successfully classified several vascular cell–type clusters (procambium, cambium, xylem and phloem), which almost completely correspond to the in vivo transcriptome data. Moreover, 346 genes were identified in the cambium cluster, among which *BEH3* was identified as a stabilizer of vascular stem cells (Furuya et al. 2021). Shi et al. obtained region-specific transcriptome data from plant stems using the fluorescence-activated nucleus sorting of various tissue-specific reporter lines and laser-captured microdissection. This region-specific analysis revealed the different signatures of the proximal cambium (xylem side) and the distal cambium (phloem side) (Shi et al. 2021). These available data sets will help to elucidate not only the complex gene regulatory networks but also the mechanisms underlying the fate determination of vascular stem cells.

## De Novo Formation of Vascular Stem Cells during Plant Regeneration and In Vitro Culture

**Fig. 4:**
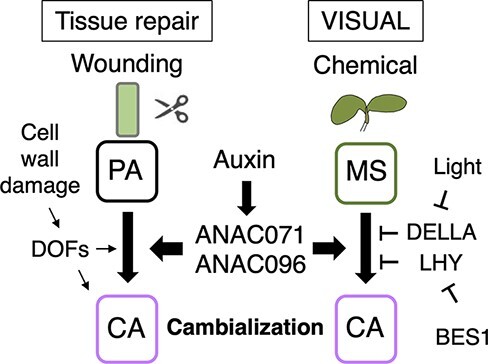
Comparison of de novo vascular stem cell formation between the tissue repair process and VISUAL. PA: parenchyma cells, MS: mesophyll cells, CA: cambium-like cells.

When the continuity of a vascular network is interrupted owing to an injury, plants respond quickly to regenerate and repair the severed tissues. After the tissue injury, auxin originating from the shoot apex accumulates above the damaged site, because of attenuating auxin downward flow. The accumulated auxin creates a new route to flow while bypassing the wound site, according to the auxin canalization hypothesis (Mazur et al. 2016). Notably, auxin-induced Arabidopsis NAC (ANAC) transcription factors, ANAC071, ANAC096 and ANAC011, play important roles in de novo vascular formation to repair the wounded vascular tissues (**Fig. 4**). Parenchyma cells close to the wound site transdifferentiate into vascular stem cell–like cells by activating the above-mentioned ANAC transcription factors, a process called cambialization (Asahina et al. 2011, Matsuoka et al. 2016). In addition to these ANACs, four vascular tissue–expressed *DOF* transcription factor genes, which are induced through the perception of wounding-induced changes in cell wall components such as cellulose and pectin, contribute to vascular reconnection as well as callus formation during tissue repair (Zhang et al. 2022). Additionally, in VISUAL, both *ANAC071* and *ANAC096* were expressed prior to the formation of vascular stem cell–like cells, and the expression of the cambium marker gene *TDR* significantly decreased in the *anac071 anac096 anac011* triple mutant (Matsuoka et al. 2021). These results suggest that vascular stem cell formation in VISUAL resembles the cambialization process that occurs during vascular reconnection (**Fig. 4**). Considering the similarities between these processes, VISUAL might be useful for understanding de novo vascular stem cell formation. Physiological analysis in the early steps of VISUAL revealed the importance of light as a signal for vascular stem cell formation (Yamazaki et al. 2018). This effect of light can be replaced by gibberellic acid treatment and is blocked by the overexpression of the constitutively active form of DELLA, suggesting that light-mediated gibberellic acid signaling is important for de novo vascular stem cell formation in VISUAL (**Fig. 4**) (Yamazaki et al. 2018).

A recent study showed that circadian clock reconstruction is involved in de novo vascular stem cell formation in VISUAL (Torii et al. 2022). The authors performed single-cell RNA-seq of samples collected at 3-h intervals during the VISUAL and conducted pseudo-time analysis based on a new algorithm, PeakMatch, in which temporal information is obtained from bulk time-course transcriptome data (Torii et al. 2022). The results revealed central clock genes such as *CIRCADIAN CLOCK ASSOCIATED 1* and *LATE ELONGATED HYPOCOTYL* (*LHY*), which are highly expressed in mesophyll cells, were gradually downregulated, whereas the evening complex-related clock genes such as *EARLY FLOWERING 3* and *LUX ARRHYTHMO* (*LUX*), which are highly expressed in vascular cells, were upregulated by the onset of VISUAL differentiation. This clock gene reconstruction is caused by the direct suppression of the *LHY* expression by BES1 (**Fig. 4**). The *bes1* mutant exhibited delayed procambium/cambium formation in addition to less vascular stem cell differentiation in VISUAL, suggesting the potential role of BES1 in de novo vascular stem cell formation (Torii et al. 2022). Moreover, a recent single-cell RNA-seq analysis of 3-day-old cotyledons revealed that *CYCLING DOF FACTOR 5* (*CDF5*) is involved in the early development of leaf veins (Liu et al. 2022). CDF5 is an important regulator of circadian rhythm and flowering time (Henriques et al. 2017, Martín et al. 2020) and is involved in the regulation of the expression of several known vascular-related genes such as *BASIC LEUCINE ZIPPER 9*, *SWEET11*/*SWEET12* and *SULFATE TRANSPORTER 2;1* (Liu et al. 2022). These data suggest that the reconstruction and modulation of the circadian clock play important roles in de novo vascular stem cell formation. Further studies will be needed to uncover the similarities and differences between the establishment and de novo formation of vascular stem cells.

## Perspective

Stem cells in the meristem are precisely maintained by neighboring cells that together form a niche. Numerous factors regulating the establishment and maintenance of vascular stem cells have been identified in previous studies, as described earlier. Plant hormones and mobile transcription factors play important roles in controlling vascular stem cells as tools for cell–cell interactions. Yet, it remains unclear where and when the cell–cell interactions occur, and spatiotemporal analyses are needed to address this unknown. Spatially resolved transcriptomic approaches such as Slide-seq have recently been developed to obtain transcriptome data linked to the positional information in a cross-section (Ståhl et al. 2016, Larsson et al. 2021, Moreno-Villena et al. 2022). Moreover, Live-seq enables the sequential transcriptome profiling of the same animal cells via cytoplasmic biopsy (Chen et al. 2022). In addition, a luminescence imaging system using a luciferase reporter can be used to successfully monitor the expression dynamics of two genes in VISUAL (Shimadzu et al. 2022). These new techniques could be used to analyze the dynamics of cell–cell interactions regulating vascular stem cells, with a good spatiotemporal resolution.

Downstream of cell–cell signaling, gene regulatory networks controlling vascular development are also being unraveled. VASCULAR-RELATED NAC DOMAIN 6 (VND6)/VND7 and APL have been identified as the master regulators of xylem and phloem differentiation, respectively (Bonke et al. 2003, Kubo et al. 2005). Recent studies on cell differentiation at the single-cell level revealed the regulatory network centered on these master regulators (Turco et al. 2019, Kamon and Ohtani 2021, Roszak et al. 2021). However, mechanisms underlying the fate specification of vascular stem cells prior to differentiation have not been revealed. Since vascular stem cells give rise to two totally distinct cell types (xylem and phloem), it seems reasonable to speculate that epigenetic regulation also contributes to cell fate determination. The application of chromatin immunoprecipitation sequencing or the assay for transposase-accessible chromatin with sequencing, together with VISUAL, can uncover the dynamics of changes in epigenetic status, thus enhancing our understanding of the stemness, i.e. the multipotency and self-renewal competency, of vascular stem cells.

## Data Availability

No new data sets were generated or analyzed in this study.
