# Correction to: Molecular Mechanisms Underlying the Establishment and Maintenance of Vascular Stem Cells in *Arabidopsis thaliana*

**DOI:** 10.1093/pcp/pcad010

**Published:** 2023-02-02

This is a correction to: Shunji Shimadzu, Tomoyuki Furuya, Yuki Kondo, Molecular Mechanisms Underlying the Establishment and Maintenance of Vascular Stem Cells in *Arabidopsis thaliana, Plant and Cell Physiology*, 2022, https://doi.org/10.1093/pcp/pcac161

In the original published version of the article, funding information for Open Access publication by KAKENHI grant number 22H04904 was omitted. This information has now been included in the online version, as below:

## Funding

Ministry of Education, Culture, Sports, Science, and Technology of Japan (17H06476 and 22H04720 to Y.K.); Japan Society for the Promotion of Science (20K15815 and 22H02647 to Y.K.); Open Access publication funded by KAKENHI grant number 22H04904.

